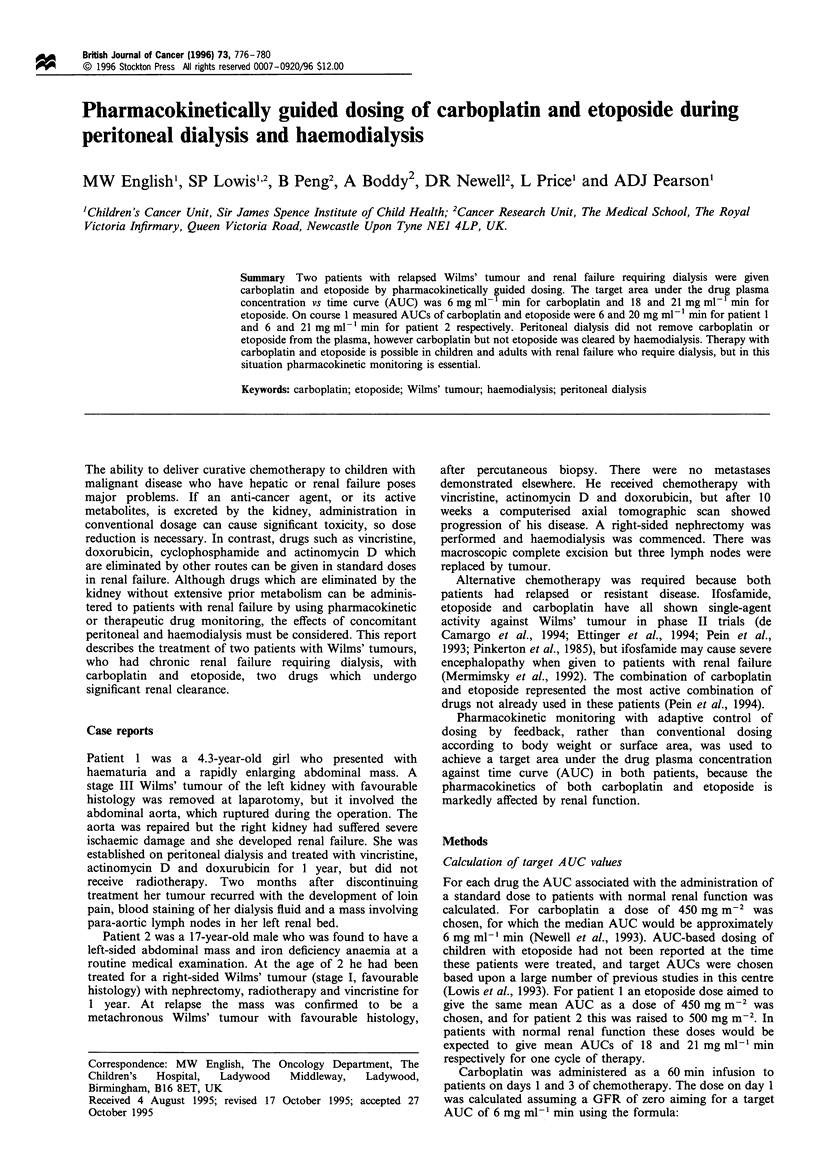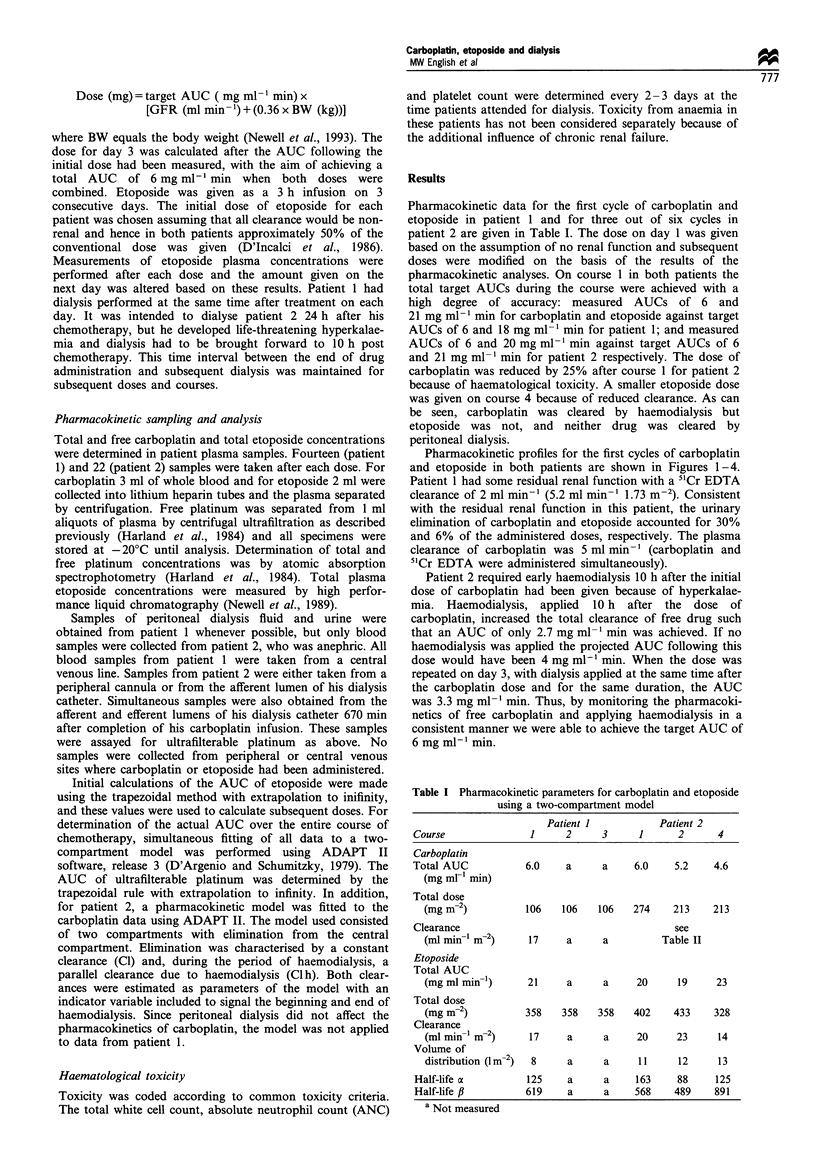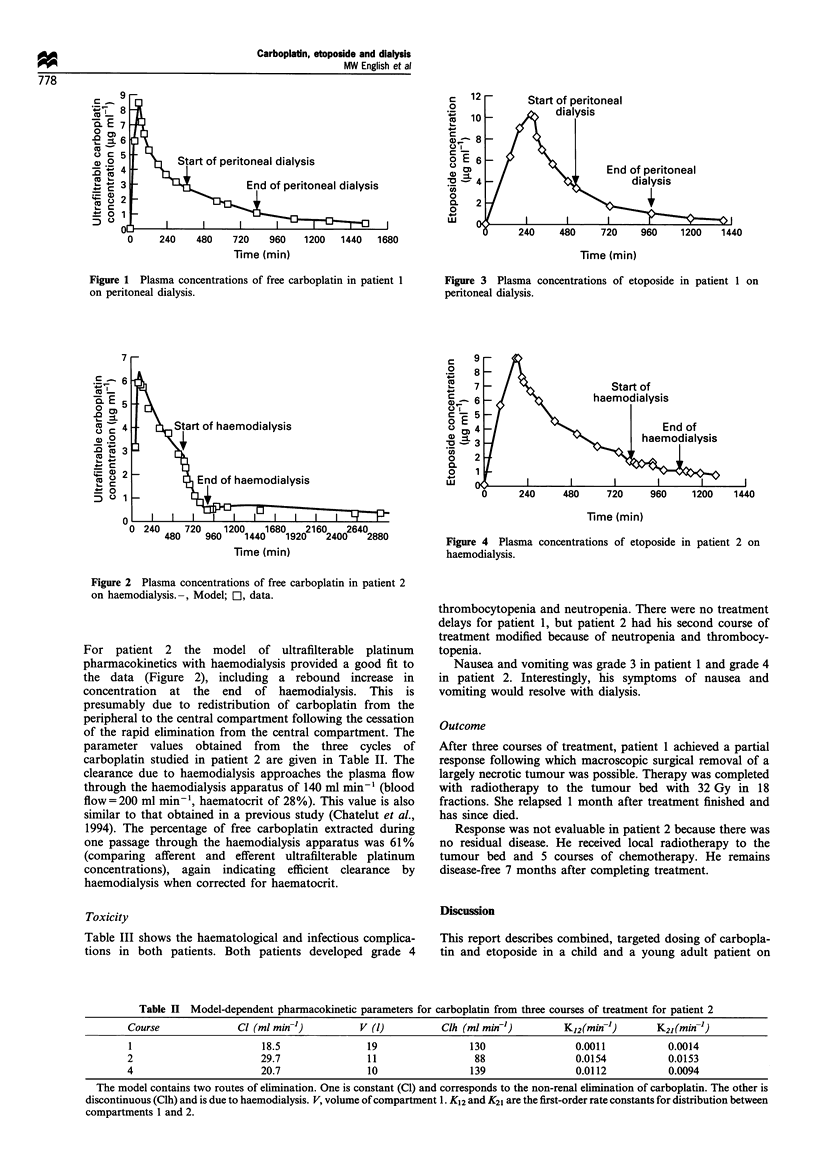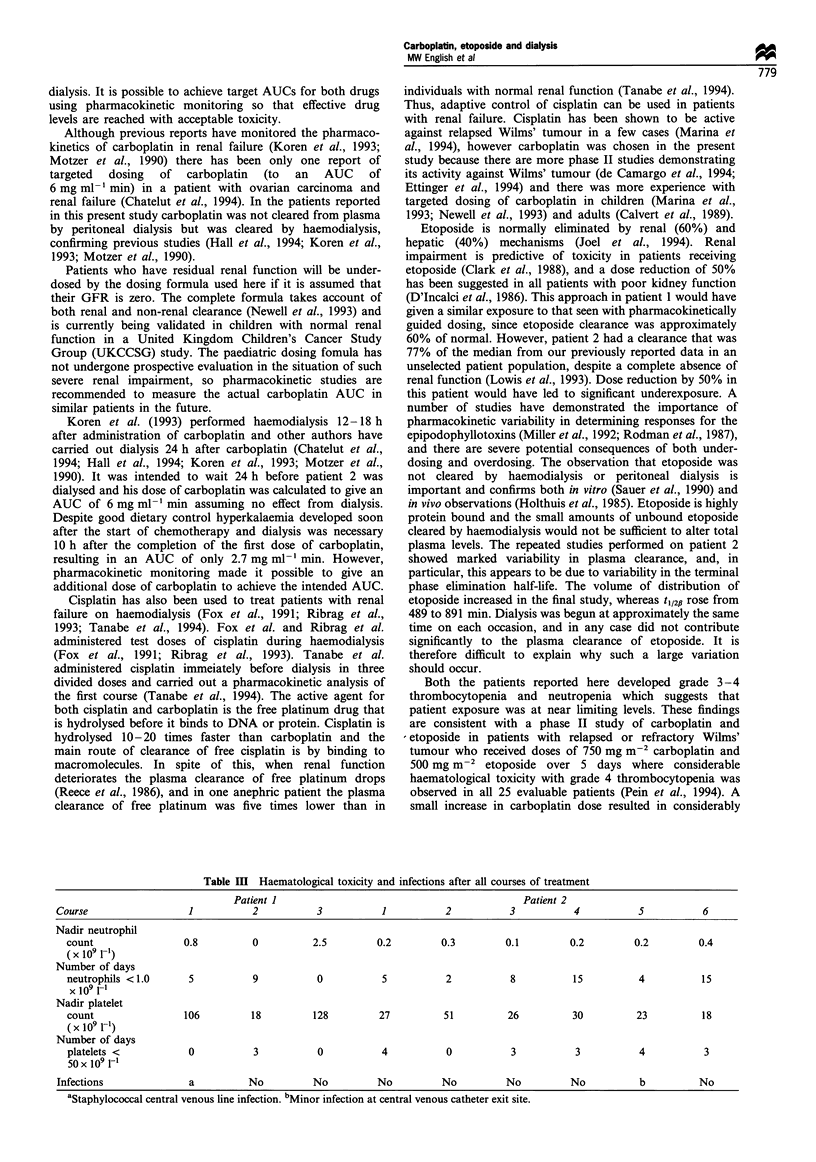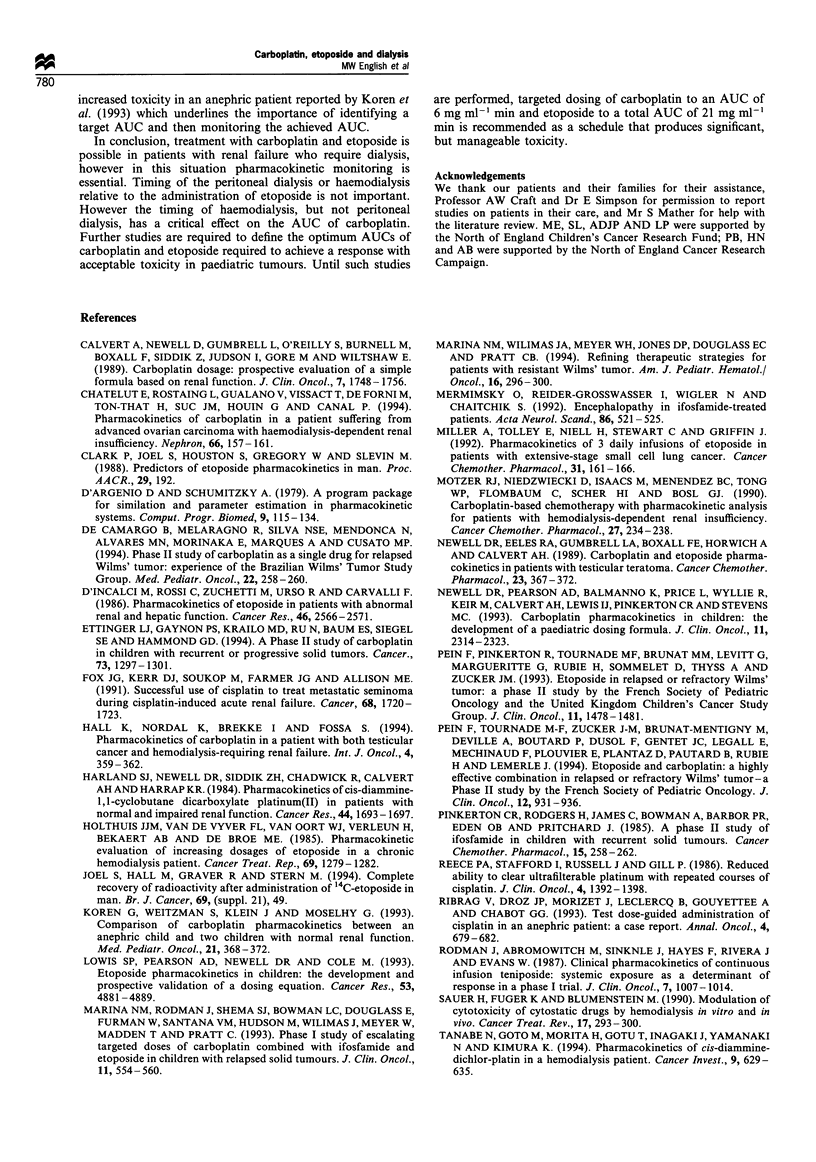# Pharmacokinetically guided dosing of carboplatin and etoposide during peritoneal dialysis and haemodialysis.

**DOI:** 10.1038/bjc.1996.135

**Published:** 1996-03

**Authors:** M. W. English, S. P. Lowis, B. Peng, A. Boddy, D. R. Newell, L. Price, A. D. Pearson

**Affiliations:** Children's cancer Unit, Sir James Spence Institute of Child Health, Cancer Research Unit, The Medical School, Newcastle Upon Tyne, UK.

## Abstract

Two patients with relapsed Wilms' tumour and renal failure requiring dialysis were given carboplatin and etoposide by pharmacokinetically guided dosing. The target area under the drug plasma concentration vs time curve (AUC) was 6 mg ml-1 min for carboplatin and 18 and 21 mg ml-1 min for etoposide. On course 1 measured AUCs of carboplatin and etoposide were 6 and 20 mg ml-1 min for patient 1 and 6 and 21 mg ml-1 min for patient 2 respectively. Peritoneal dialysis did not remove carboplatin or etoposide from the plasma, however carboplatin but not etoposide was cleared by haemodialysis. Therapy with carboplatin and etoposide is possible in children and adults with renal failure who require dialysis, but in this situation pharmacokinetic monitoring is essential.


British Journal of Cancer (1996) 73, 776-780
© 1996 Stockton Press All rights reserved 0007-0920/96 $12.00

### Pharmacokinetically guided dosing of carboplatin and etoposide during peritoneal dialysis and haemodialysis

MW English[1], SP Lowis[1,2], B Peng[2], A Boddy[2], DR Newell[2], L Price[1] and ADJ Pearson[1]

[1]*Children's Cancer Unit, Sir James Spence Institute of Child Health;* [2]*Cancer Research Unit, The Medical School, The Royal Victoria Infirmary, Queen Victoria Road, Newcastle Upon Tyne NE1 4LP, UK.*

**Summary** Two patients with relapsed Wilms' tumour and renal failure requiring dialysis were given carboplatin and etoposide by pharmacokinetically guided dosing. The target area under the drug plasma concentration *vs* time curve (AUC) was 6 mg ml$^{-1}$ min for carboplatin and 18 and 21 mg ml$^{-1}$ min for etoposide. On course 1 measured AUCs of carboplatin and etoposide were 6 and 20 mg ml$^{-1}$ min for patient 1 and 6 and 21 mg ml$^{-1}$ min for patient 2 respectively. Peritoneal dialysis did not remove carboplatin or etoposide from the plasma, however carboplatin but not etoposide was cleared by haemodialysis. Therapy with carboplatin and etoposide is possible in children and adults with renal failure who require dialysis, but in this situation pharmacokinetic monitoring is essential.

**Keywords:** carboplatin; etoposide; Wilms' tumour; haemodialysis; peritoneal dialysis

The ability to deliver curative chemotherapy to children with malignant disease who have hepatic or renal failure poses major problems. If an anti-cancer agent, or its active metabolites, is excreted by the kidney, administration in conventional dosage can cause significant toxicity, so dose reduction is necessary. In contrast, drugs such as vincristine, doxorubicin, cyclophosphamide and actinomycin D which are eliminated by other routes can be given in standard doses in renal failure. Although drugs which are eliminated by the kidney without extensive prior metabolism can be administered to patients with renal failure by using pharmacokinetic or therapeutic drug monitoring, the effects of concomitant peritoneal and haemodialysis must be considered. This report describes the treatment of two patients with Wilms' tumours, who had chronic renal failure requiring dialysis, with carboplatin and etoposide, two drugs which undergo significant renal clearance.

### Case reports

Patient 1 was a 4.3-year-old girl who presented with haematuria and a rapidly enlarging abdominal mass. A stage III Wilms' tumour of the left kidney with favourable histology was removed at laparotomy, but it involved the abdominal aorta, which ruptured during the operation. The aorta was repaired but the right kidney had suffered severe ischaemic damage and she developed renal failure. She was established on peritoneal dialysis and treated with vincristine, actinomycin D and doxurubicin for 1 year, but did not receive radiotherapy. Two months after discontinuing treatment her tumour recurred with the development of loin pain, blood staining of her dialysis fluid and a mass involving para-aortic lymph nodes in her left renal bed.

Patient 2 was a 17-year-old male who was found to have a left-sided abdominal mass and iron deficiency anaemia at a routine medical examination. At the age of 2 he had been treated for a right-sided Wilms' tumour (stage I, favourable histology) with nephrectomy, radiotherapy and vincristine for 1 year. At relapse the mass was confirmed to be a metachronous Wilms' tumour with favourable histology, after percutaneous biopsy. There were no metastases demonstrated elsewhere. He received chemotherapy with vincristine, actinomycin D and doxorubicin, but after 10 weeks a computerised axial tomographic scan showed progression of his disease. A right-sided nephrectomy was performed and haemodialysis was commenced. There was macroscopic complete excision but three lymph nodes were replaced by tumour.

Alternative chemotherapy was required because both patients had relapsed or resistant disease. Ifosfamide, etoposide and carboplatin have all shown single-agent activity against Wilms' tumour in phase II trials (de Camargo *et al.*, 1994; Ettinger *et al.*, 1994; Pein *et al.*, 1993; Pinkerton *et al.*, 1985), but ifosfamide may cause severe encephalopathy when given to patients with renal failure (Mermimsky *et al.*, 1992). The combination of carboplatin and etoposide represented the most active combination of drugs not already used in these patients (Pein *et al.*, 1994).

Pharmacokinetic monitoring with adaptive control of dosing by feedback, rather than conventional dosing according to body weight or surface area, was used to achieve a target area under the drug plasma concentration against time curve (AUC) in both patients, because the pharmacokinetics of both carboplatin and etoposide is markedly affected by renal function.

### Methods

### *Calculation of target AUC values*

For each drug the AUC associated with the administration of a standard dose to patients with normal renal function was calculated. For carboplatin a dose of 450 mg m$^{-2}$ was chosen, for which the median AUC would be approximately 6 mg ml$^{-1}$ min (Newell *et al.*, 1993). AUC-based dosing of children with etoposide had not been reported at the time these patients were treated, and target AUCs were chosen based upon a large number of previous studies in this centre (Lowis *et al.*, 1993). For patient 1 an etoposide dose aimed to give the same mean AUC as a dose of 450 mg m$^{-2}$ was chosen, and for patient 2 this was raised to 500 mg m$^{-2}$. In patients with normal renal function these doses would be expected to give mean AUCs of 18 and 21 mg ml$^{-1}$ min respectively for one cycle of therapy.

Carboplatin was administered as a 60 min infusion to patients on days 1 and 3 of chemotherapy. The dose on day 1 was calculated assuming a GFR of zero aiming for a target AUC of 6 mg ml$^{-1}$ min using the formula:

Correspondence: MW English, The Oncology Department, The Children's Hospital, Ladywood Middleway, Ladywood, Birmingham, B16 8ET, UK
Received 4 August 1995; revised 17 October 1995; accepted 27 October 1995

$$\text{Dose (mg)} = \text{target AUC (mg ml}^{-1}\text{ min)} \times [\text{GFR (ml min}^{-1}) + (0.36 \times \text{BW (kg)})]$$

where BW equals the body weight (Newell *et al.*, 1993). The dose for day 3 was calculated after the AUC following the initial dose had been measured, with the aim of achieving a total AUC of 6 mg ml$^{-1}$ min when both doses were combined. Etoposide was given as a 3 h infusion on 3 consecutive days. The initial dose of etoposide for each patient was chosen assuming that all clearance would be non-renal and hence in both patients approximately 50% of the conventional dose was given (D'Incalci *et al.*, 1986). Measurements of etoposide plasma concentrations were performed after each dose and the amount given on the next day was altered based on these results. Patient 1 had dialysis performed at the same time after treatment on each day. It was intended to dialyse patient 2 24 h after his chemotherapy, but he developed life-threatening hyperkalaemia and dialysis had to be brought forward to 10 h post chemotherapy. This time interval between the end of drug administration and subsequent dialysis was maintained for subsequent doses and courses.

*Pharmacokinetic sampling and analysis*

Total and free carboplatin and total etoposide concentrations were determined in patient plasma samples. Fourteen (patient 1) and 22 (patient 2) samples were taken after each dose. For carboplatin 3 ml of whole blood and for etoposide 2 ml were collected into lithium heparin tubes and the plasma separated by centrifugation. Free platinum was separated from 1 ml aliquots of plasma by centrifugal ultrafiltration as described previously (Harland *et al.*, 1984) and all specimens were stored at −20°C until analysis. Determination of total and free platinum concentrations was by atomic absorption spectrophotometry (Harland *et al.*, 1984). Total plasma etoposide concentrations were measured by high performance liquid chromatography (Newell *et al.*, 1989).

Samples of peritoneal dialysis fluid and urine were obtained from patient 1 whenever possible, but only blood samples were collected from patient 2, who was anephric. All blood samples from patient 1 were taken from a central venous line. Samples from patient 2 were either taken from a peripheral cannula or from the afferent lumen of his dialysis catheter. Simultaneous samples were also obtained from the afferent and efferent lumens of his dialysis catheter 670 min after completion of his carboplatin infusion. These samples were assayed for ultrafilterable platinum as above. No samples were collected from peripheral or central venous sites where carboplatin or etoposide had been administered.

Initial calculations of the AUC of etoposide were made using the trapezoidal method with extrapolation to inifinity, and these values were used to calculate subsequent doses. For determination of the actual AUC over the entire course of chemotherapy, simultaneous fitting of all data to a two-compartment model was performed using ADAPT II software, release 3 (D'Argenio and Schumitzky, 1979). The AUC of ultrafilterable platinum was determined by the trapezoidal rule with extrapolation to infinity. In addition, for patient 2, a pharmacokinetic model was fitted to the carboplatin data using ADAPT II. The model used consisted of two compartments with elimination from the central compartment. Elimination was characterised by a constant clearance (Cl) and, during the period of haemodialysis, a parallel clearance due to haemodialysis (Cl h). Both clearances were estimated as parameters of the model with an indicator variable included to signal the beginning and end of haemodialysis. Since peritoneal dialysis did not affect the pharmacokinetics of carboplatin, the model was not applied to data from patient 1.

*Haematological toxicity*

Toxicity was coded according to common toxicity criteria. The total white cell count, absolute neutrophil count (ANC) and platelet count were determined every 2–3 days at the time patients attended for dialysis. Toxicity from anaemia in these patients has not been considered separately because of the additional influence of chronic renal failure.

### Results

Pharmacokinetic data for the first cycle of carboplatin and etoposide in patient 1 and for three out of six cycles in patient 2 are given in Table I. The dose on day 1 was given based on the assumption of no renal function and subsequent doses were modified on the basis of the results of the pharmacokinetic analyses. On course 1 in both patients the total target AUCs during the course were achieved with a high degree of accuracy: measured AUCs of 6 and 21 mg ml$^{-1}$ min for carboplatin and etoposide against target AUCs of 6 and 18 mg ml$^{-1}$ min for patient 1; and measured AUCs of 6 and 20 mg ml$^{-1}$ min against target AUCs of 6 and 21 mg ml$^{-1}$ min for patient 2 respectively. The dose of carboplatin was reduced by 25% after course 1 for patient 2 because of haematological toxicity. A smaller etoposide dose was given on course 4 because of reduced clearance. As can be seen, carboplatin was cleared by haemodialysis but etoposide was not, and neither drug was cleared by peritoneal dialysis.

Pharmacokinetic profiles for the first cycles of carboplatin and etoposide in both patients are shown in Figures 1–4. Patient 1 had some residual renal function with a $^{51}$Cr EDTA clearance of 2 ml min$^{-1}$ (5.2 ml min$^{-1}$ 1.73 m$^{-2}$). Consistent with the residual renal function in this patient, the urinary elimination of carboplatin and etoposide accounted for 30% and 6% of the administered doses, respectively. The plasma clearance of carboplatin was 5 ml min$^{-1}$ (carboplatin and $^{51}$Cr EDTA were administered simultaneously).

Patient 2 required early haemodialysis 10 h after the initial dose of carboplatin had been given because of hyperkalaemia. Haemodialysis, applied 10 h after the dose of carboplatin, increased the total clearance of free drug such that an AUC of only 2.7 mg ml$^{-1}$ min was achieved. If no haemodialysis was applied the projected AUC following this dose would have been 4 mg ml$^{-1}$ min. When the dose was repeated on day 3, with dialysis applied at the same time after the carboplatin dose and for the same duration, the AUC was 3.3 mg ml$^{-1}$ min. Thus, by monitoring the pharmacokinetics of free carboplatin and applying haemodialysis in a consistent manner we were able to achieve the target AUC of 6 mg ml$^{-1}$ min.

**Table I** Pharmacokinetic parameters for carboplatin and etoposide using a two-compartment model

| | *Patient 1* | | | *Patient 2* | | |
|---|---|---|---|---|---|---|
| *Course* | *1* | *2* | *3* | *1* | *2* | *4* |
| *Carboplatin* | | | | | | |
| Total AUC (mg ml$^{-1}$ min) | 6.0 | a | a | 6.0 | 5.2 | 4.6 |
| Total dose (mg m$^{-2}$) | 106 | 106 | 106 | 274 | 213 | 213 |
| Clearance (ml min$^{-1}$ m$^{-2}$) | 17 | a | a | | see Table II | |
| *Etoposide* | | | | | | |
| Total AUC (mg ml min$^{-1}$) | 21 | a | a | 20 | 19 | 23 |
| Total dose (mg m$^{-2}$) | 358 | 358 | 358 | 402 | 433 | 328 |
| Clearance (ml min$^{-1}$ m$^{-2}$) | 17 | a | a | 20 | 23 | 14 |
| Volume of distribution (l m$^{-2}$) | 8 | a | a | 11 | 12 | 13 |
| Half-life α | 125 | a | a | 163 | 88 | 125 |
| Half-life β | 619 | a | a | 568 | 489 | 891 |

[a] Not measured

**Figure 1** Plasma concentrations of free carboplatin in patient 1 on peritoneal dialysis.

**Figure 3** Plasma concentrations of etoposide in patient 1 on peritoneal dialysis.

**Figure 2** Plasma concentrations of free carboplatin in patient 2 on haemodialysis.–, Model; □, data.

**Figure 4** Plasma concentrations of etoposide in patient 2 on haemodialysis.

For patient 2 the model of ultrafilterable platinum pharmacokinetics with haemodialysis provided a good fit to the data (Figure 2), including a rebound increase in concentration at the end of haemodialysis. This is presumably due to redistribution of carboplatin from the peripheral to the central compartment following the cessation of the rapid elimination from the central compartment. The parameter values obtained from the three cycles of carboplatin studied in patient 2 are given in Table II. The clearance due to haemodialysis approaches the plasma flow through the haemodialysis apparatus of 140 ml min$^{-1}$ (blood flow = 200 ml min$^{-1}$, haematocrit of 28%). This value is also similar to that obtained in a previous study (Chatelut *et al.*, 1994). The percentage of free carboplatin extracted during one passage through the haemodialysis apparatus was 61% (comparing afferent and efferent ultrafilterable platinum concentrations), again indicating efficient clearance by haemodialysis when corrected for haematocrit.

### *Toxicity*

Table III shows the haematological and infectious complications in both patients. Both patients developed grade 4 thrombocytopenia and neutropenia. There were no treatment delays for patient 1, but patient 2 had his second course of treatment modified because of neutropenia and thrombocytopenia.

Nausea and vomiting was grade 3 in patient 1 and grade 4 in patient 2. Interestingly, his symptoms of nausea and vomiting would resolve with dialysis.

### *Outcome*

After three courses of treatment, patient 1 achieved a partial response following which macroscopic surgical removal of a largely necrotic tumour was possible. Therapy was completed with radiotherapy to the tumour bed with 32 Gy in 18 fractions. She relapsed 1 month after treatment finished and has since died.

Response was not evaluable in patient 2 because there was no residual disease. He received local radiotherapy to the tumour bed and 5 courses of chemotherapy. He remains disease-free 7 months after completing treatment.

### Discussion

This report describes combined, targeted dosing of carboplatin and etoposide in a child and a young adult patient on

**Table II** Model-dependent pharmacokinetic parameters for carboplatin from three courses of treatment for patient 2

| *Course* | *Cl (ml min$^{-1}$)* | *V (l)* | *Clh (ml min$^{-1}$)* | *$K_{12}$(min$^{-1}$)* | *$K_{21}$(min$^{-1}$)* |
|---|---|---|---|---|---|
| 1 | 18.5 | 19 | 130 | 0.0011 | 0.0014 |
| 2 | 29.7 | 11 | 88 | 0.0154 | 0.0153 |
| 4 | 20.7 | 10 | 139 | 0.0112 | 0.0094 |

The model contains two routes of elimination. One is constant (Cl) and corresponds to the non-renal elimination of carboplatin. The other is discontinuous (Clh) and is due to haemodialysis. *V*, volume of compartment 1. $K_{12}$ and $K_{21}$ are the first-order rate constants for distribution between compartments 1 and 2.

dialysis. It is possible to achieve target AUCs for both drugs using pharmacokinetic monitoring so that effective drug levels are reached with acceptable toxicity.

Although previous reports have monitored the pharmacokinetics of carboplatin in renal failure (Koren *et al.*, 1993; Motzer *et al.*, 1990) there has been only one report of targeted dosing of carboplatin (to an AUC of 6 mg $ml^{-1}$ min) in a patient with ovarian carcinoma and renal failure (Chatelut *et al.*, 1994). In the patients reported in this present study carboplatin was not cleared from plasma by peritoneal dialysis but was cleared by haemodialysis, confirming previous studies (Hall *et al.*, 1994; Koren *et al.*, 1993; Motzer *et al.*, 1990).

Patients who have residual renal function will be underdosed by the dosing formula used here if it is assumed that their GFR is zero. The complete formula takes account of both renal and non-renal clearance (Newell *et al.*, 1993) and is currently being validated in children with normal renal function in a United Kingdom Children's Cancer Study Group (UKCCSG) study. The paediatric dosing fomula has not undergone prospective evaluation in the situation of such severe renal impairment, so pharmacokinetic studies are recommended to measure the actual carboplatin AUC in similar patients in the future.

Koren *et al.* (1993) performed haemodialysis 12–18 h after administration of carboplatin and other authors have carried out dialysis 24 h after carboplatin (Chatelut *et al.*, 1994; Hall *et al.*, 1994; Koren *et al.*, 1993; Motzer *et al.*, 1990). It was intended to wait 24 h before patient 2 was dialysed and his dose of carboplatin was calculated to give an AUC of 6 mg $ml^{-1}$ min assuming no effect from dialysis. Despite good dietary control hyperkalaemia developed soon after the start of chemotherapy and dialysis was necessary 10 h after the completion of the first dose of carboplatin, resulting in an AUC of only 2.7 mg $ml^{-1}$ min. However, pharmacokinetic monitoring made it possible to give an additional dose of carboplatin to achieve the intended AUC.

Cisplatin has also been used to treat patients with renal failure on haemodialysis (Fox *et al.*, 1991; Ribrag *et al.*, 1993; Tanabe *et al.*, 1994). Fox *et al.* and Ribrag *et al.* administered test doses of cisplatin during haemodialysis (Fox *et al.*, 1991; Ribrag *et al.*, 1993). Tanabe *et al.* administered cisplatin immeiately before dialysis in three divided doses and carried out a pharmacokinetic analysis of the first course (Tanabe *et al.*, 1994). The active agent for both cisplatin and carboplatin is the free platinum drug that is hydrolysed before it binds to DNA or protein. Cisplatin is hydrolysed 10–20 times faster than carboplatin and the main route of clearance of free cisplatin is by binding to macromolecules. In spite of this, when renal function deteriorates the plasma clearance of free platinum drops (Reece *et al.*, 1986), and in one anephric patient the plasma clearance of free platinum was five times lower than in individuals with normal renal function (Tanabe *et al.*, 1994). Thus, adaptive control of cisplatin can be used in patients with renal failure. Cisplatin has been shown to be active against relapsed Wilms' tumour in a few cases (Marina *et al.*, 1994), however carboplatin was chosen in the present study because there are more phase II studies demonstrating its activity against Wilms' tumour (de Camargo *et al.*, 1994; Ettinger *et al.*, 1994) and there was more experience with targeted dosing of carboplatin in children (Marina *et al.*, 1993; Newell *et al.*, 1993) and adults (Calvert *et al.*, 1989).

Etoposide is normally eliminated by renal (60%) and hepatic (40%) mechanisms (Joel *et al.*, 1994). Renal impairment is predictive of toxicity in patients receiving etoposide (Clark *et al.*, 1988), and a dose reduction of 50% has been suggested in all patients with poor kidney function (D'Incalci *et al.*, 1986). This approach in patient 1 would have given a similar exposure to that seen with pharmacokinetically guided dosing, since etoposide clearance was approximately 60% of normal. However, patient 2 had a clearance that was 77% of the median from our previously reported data in an unselected patient population, despite a complete absence of renal function (Lowis *et al.*, 1993). Dose reduction by 50% in this patient would have led to significant underexposure. A number of studies have demonstrated the importance of pharmacokinetic variability in determining responses for the epipodophyllotoxins (Miller *et al.*, 1992; Rodman *et al.*, 1987), and there are severe potential consequences of both underdosing and overdosing. The observation that etoposide was not cleared by haemodialysis or peritoneal dialysis is important and confirms both *in vitro* (Sauer *et al.*, 1990) and *in vivo* observations (Holthuis *et al.*, 1985). Etoposide is highly protein bound and the small amounts of unbound etoposide cleared by haemodialysis would not be sufficient to alter total plasma levels. The repeated studies performed on patient 2 showed marked variability in plasma clearance, and, in particular, this appears to be due to variability in the terminal phase elimination half-life. The volume of distribution of etoposide increased in the final study, whereas $t_{1/2\beta}$ rose from 489 to 891 min. Dialysis was begun at approximately the same time on each occasion, and in any case did not contribute significantly to the plasma clearance of etoposide. It is therefore difficult to explain why such a large variation should occur.

Both the patients reported here developed grade 3–4 thrombocytopenia and neutropenia which suggests that patient exposure was at near limiting levels. These findings are consistent with a phase II study of carboplatin and etoposide in patients with relapsed or refractory Wilms' tumour who received doses of 750 mg $m^{-2}$ carboplatin and 500 mg $m^{-2}$ etoposide over 5 days where considerable haematological toxicity with grade 4 thrombocytopenia was observed in all 25 evaluable patients (Pein *et al.*, 1994). A small increase in carboplatin dose resulted in considerably

**Table III** Haematological toxicity and infections after all courses of treatment

| | Patient 1 | | | Patient 2 | | | | | |
|---|---|---|---|---|---|---|---|---|---|
| *Course* | *1* | *2* | *3* | *1* | *2* | *3* | *4* | *5* | *6* |
| Nadir neutrophil count ($\times 10^9 l^{-1}$) | 0.8 | 0 | 2.5 | 0.2 | 0.3 | 0.1 | 0.2 | 0.2 | 0.4 |
| Number of days neutrophils <1.0 $\times 10^9 l^{-1}$ | 5 | 9 | 0 | 5 | 2 | 8 | 15 | 4 | 15 |
| Nadir platelet count ($\times 10^9 l^{-1}$) | 106 | 18 | 128 | 27 | 51 | 26 | 30 | 23 | 18 |
| Number of days platelets < $50 \times 10^9 l^{-1}$ | 0 | 3 | 0 | 4 | 0 | 3 | 3 | 4 | 3 |
| Infections | a | No | No | No | No | No | No | b | No |

[a]Staphylococcal central venous line infection. [b]Minor infection at central venous catheter exit site.

increased toxicity in an anephric patient reported by Koren *et al.* (1993) which underlines the importance of identifying a target AUC and then monitoring the achieved AUC.

In conclusion, treatment with carboplatin and etoposide is possible in patients with renal failure who require dialysis, however in this situation pharmacokinetic monitoring is essential. Timing of the peritoneal dialysis or haemodialysis relative to the administration of etoposide is not important. However the timing of haemodialysis, but not peritoneal dialysis, has a critical effect on the AUC of carboplatin. Further studies are required to define the optimum AUCs of carboplatin and etoposide required to achieve a response with acceptable toxicity in paediatric tumours. Until such studies are performed, targeted dosing of carboplatin to an AUC of 6 mg $ml^{-1}$ min and etoposide to a total AUC of 21 mg $ml^{-1}$ min is recommended as a schedule that produces significant, but manageable toxicity.

**Acknowledgements**
We thank our patients and their families for their assistance, Professor AW Craft and Dr E Simpson for permission to report studies on patients in their care, and Mr S Mather for help with the literature review. ME, SL, ADJP AND LP were supported by the North of England Children's Cancer Research Fund; PB, HN and AB were supported by the North of England Cancer Research Campaign.

### References

CALVERT A, NEWELL D, GUMBRELL L, O'REILLY S, BURNELL M, BOXALL F, SIDDIK Z, JUDSON I, GORE M AND WILTSHAW E. (1989). Carboplatin dosage: prospective evaluation of a simple formula based on renal function. *J. Clin. Oncol.*, **7**, 1748–1756.

CHATELUT E, ROSTAING L, GUALANO V, VISSACT T, DE FORNI M, TON-THAT H, SUC JM, HOUIN G AND CANAL P. (1994). Pharmacokinetics of carboplatin in a patient suffering from advanced ovarian carcinoma with haemodialysis-dependent renal insufficiency. *Nephron*, **66**, 157–161.

CLARK P, JOEL S, HOUSTON S, GREGORY W AND SLEVIN M. (1988). Predictors of etoposide pharmacokinetics in man. *Proc. AACR.*, **29**, 192.

D'ARGENIO D AND SCHUMITZKY A. (1979). A program package for similation and parameter estimation in pharmacokinetic systems. *Comput. Progr. Biomed*, **9**, 115–134.

DE CAMARGO B, MELARAGNO R, SILVA NSE, MENDONCA N, ALVARES MN, MORINAKA E, MARQUES A AND CUSATO MP. (1994). Phase II study of carboplatin as a single drug for relapsed Wilms' tumor: experience of the Brazilian Wilms' Tumor Study Group. *Med. Pediatr. Oncol.*, **22**, 258–260.

D'INCALCI M, ROSSI C, ZUCHETTI M, URSO R AND CARVALLI F. (1986). Pharmacokinetics of etoposide in patients with abnormal renal and hepatic function. *Cancer Res.*, **46**, 2566–2571.

ETTINGER LJ, GAYNON PS, KRAILO MD, RU N, BAUM ES, SIEGEL SE AND HAMMOND GD. (1994). A Phase II study of carboplatin in children with recurrent or progressive solid tumors. *Cancer.*, **73**, 1297–1301.

FOX JG, KERR DJ, SOUKOP M, FARMER JG AND ALLISON ME. (1991). Successful use of cisplatin to treat metastatic seminoma during cisplatin-induced acute renal failure. *Cancer*, **68**, 1720–1723.

HALL K, NORDAL K, BREKKE I AND FOSSA S. (1994). Pharmacokinetics of carboplatin in a patient with both testicular cancer and hemodialysis-requiring renal failure. *Int. J. Oncol.*, **4**, 359–362.

HARLAND SJ, NEWELL DR, SIDDIK ZH, CHADWICK R, CALVERT AH AND HARRAP KR. (1984). Pharmacokinetics of cis-diammine-1,1-cyclobutane dicarboxylate platinum(II) in patients with normal and impaired renal function. *Cancer Res.*, **44**, 1693–1697.

HOLTHUIS JJM, VAN DE VYVER FL, VAN OORT WJ, VERLEUN H, BEKAERT AB AND DE BROE ME. (1985). Pharmacokinetic evaluation of increasing dosages of etoposide in a chronic hemodialysis patient. *Cancer Treat. Rep.*, **69**, 1279–1282.

JOEL S, HALL M, GRAVER R AND STERN M. (1994). Complete recovery of radioactivity after administration of $^{14}$C-etoposide in man. *Br. J. Cancer*, **69**, (suppl. 21), 49.

KOREN G, WEITZMAN S, KLEIN J AND MOSELHY G. (1993). Comparison of carboplatin pharmacokinetics between an anephric child and two children with normal renal function. *Med. Pediatr. Oncol.*, **21**, 368–372.

LOWIS SP, PEARSON AD, NEWELL DR AND COLE M. (1993). Etoposide pharmacokinetics in children: the development and prospective validation of a dosing equation. *Cancer Res.*, **53**, 4881–4889.

MARINA NM, RODMAN J, SHEMA SJ, BOWMAN LC, DOUGLASS E, FURMAN W, SANTANA VM, HUDSON M, WILIMAS J, MEYER W, MADDEN T AND PRATT C. (1993). Phase I study of escalating targeted doses of carboplatin combined with ifosfamide and etoposide in children with relapsed solid tumours. *J. Clin. Oncol.*, **11**, 554–560.

MARINA NM, WILIMAS JA, MEYER WH, JONES DP, DOUGLASS EC AND PRATT CB. (1994). Refining therapeutic strategies for patients with resistant Wilms' tumor. *Am. J. Pediatr. Hematol./Oncol.*, **16**, 296–300.

MERMIMSKY O, REIDER-GROSSWASSER I, WIGLER N AND CHAITCHIK S. (1992). Encephalopathy in ifosfamide-treated patients. *Acta Neurol. Scand.*, **86**, 521–525.

MILLER A, TOLLEY E, NIELL H, STEWART C AND GRIFFIN J. (1992). Pharmacokinetics of 3 daily infusions of etoposide in patients with extensive-stage small cell lung cancer. *Cancer Chemother. Pharmacol.*, **31**, 161–166.

MOTZER RJ, NIEDZWIECKI D, ISAACS M, MENENDEZ BC, TONG WP, FLOMBAUM C, SCHER HI AND BOSL GJ. (1990). Carboplatin-based chemotherapy with pharmacokinetic analysis for patients with hemodialysis-dependent renal insufficiency. *Cancer Chemother. Pharmacol.*, **27**, 234–238.

NEWELL DR, EELES RA, GUMBRELL LA, BOXALL FE, HORWICH A AND CALVERT AH. (1989). Carboplatin and etoposide pharmacokinetics in patients with testicular teratoma. *Cancer Chemother. Pharmacol.*, **23**, 367–372.

NEWELL DR, PEARSON AD, BALMANNO K, PRICE L, WYLLIE R, KEIR M, CALVERT AH, LEWIS IJ, PINKERTON CR AND STEVENS MC. (1993). Carboplatin pharmacokinetics in children: the development of a paediatric dosing formula. *J. Clin. Oncol.*, **11**, 2314–2323.

PEIN F, PINKERTON R, TOURNADE MF, BRUNAT MM, LEVITT G, MARGUERITTE G, RUBIE H, SOMMELET D, THYSS A AND ZUCKER JM. (1993). Etoposide in relapsed or refractory Wilms' tumor: a phase II study by the French Society of Pediatric Oncology and the United Kingdom Children's Cancer Study Group. *J. Clin. Oncol.*, **11**, 1478–1481.

PEIN F, TOURNADE M-F, ZUCKER J-M, BRUNAT-MENTIGNY M, DEVILLE A, BOUTARD P, DUSOL F, GENTET JC, LEGALL E, MECHINAUD F, PLOUVIER E, PLANTAZ D, PAUTARD B, RUBIE H AND LEMERLE J. (1994). Etoposide and carboplatin: a highly effective combination in relapsed or refractory Wilms' tumor–a Phase II study by the French Society of Pediatric Oncology. *J. Clin. Oncol.*, **12**, 931–936.

PINKERTON CR, RODGERS H, JAMES C, BOWMAN A, BARBOR PR, EDEN OB AND PRITCHARD J. (1985). A phase II study of ifosfamide in children with recurrent solid tumours. *Cancer Chemother. Pharmacol.*, **15**, 258–262.

REECE PA, STAFFORD I, RUSSELL J AND GILL P. (1986). Reduced ability to clear ultrafilterable platinum with repeated courses of cisplatin. *J. Clin. Oncol.*, **4**, 1392–1398.

RIBRAG V, DROZ JP, MORIZET J, LECLERCQ B, GOUYETTEE A AND CHABOT GG. (1993). Test dose-guided administration of cisplatin in an anephric patient: a case report. *Annal. Oncol.*, **4**, 679–682.

RODMAN J, ABROMOWITCH M, SINKNLE J, HAYES F, RIVERA J AND EVANS W. (1987). Clinical pharmacokinetics of continuous infusion teniposide: systemic exposure as a determinant of response in a phase I trial. *J. Clin. Oncol.*, **7**, 1007–1014.

SAUER H, FUGER K AND BLUMENSTEIN M. (1990). Modulation of cytotoxicity of cytostatic drugs by hemodialysis *in vitro* and *in vivo*. *Cancer Treat. Rev.*, **17**, 293–300.

TANABE N, GOTO M, MORITA H, GOTU T, INAGAKI J, YAMANAKI N AND KIMURA K. (1994). Pharmacokinetics of *cis*-diammine-dichlor-platin in a hemodialysis patient. *Cancer Invest.*, **9**, 629–635.